# Role of rectal colonization by third-generation cephalosporin-resistant Enterobacterales on the risk of surgical site infection after hepato-pancreato-biliary surgery

**DOI:** 10.1128/spectrum.00878-24

**Published:** 2024-09-24

**Authors:** Miguel Rodríguez-Fernández, Marta Trigo-Rodríguez, Darío Martínez-Baena, Rocío Herrero, Reinaldo Espíndola-Gómez, Pedro Martínez Pérez-Crespo, Alberto Gallego Vela, Eva Torres, Ana Isabel Aller García, Eva M. León, Juan E. Corzo-Delgado, Pablo Parra-Membrives, Nicolás Merchante

**Affiliations:** 1 Unidad de Enfermedades Infecciosas y Microbiología, Hospital Universitario de Valme, Instituto de Biomedicina de Sevilla (IBiS), Universidad de Sevilla, Sevilla, Spain; 2 Unidad de Cirugía Hepato-Bilio-Pancreática, Servicio de Cirugía General, Hospital Universitario de Valme, Sevilla, Spain; JMI Laboratories, North Liberty, Iowa, USA

**Keywords:** third-generation cephalosporin-resistant Enterobacterales (3GCR-E), intestinal carriage status, hepato-pancreato-biliary resection surgery, perioperative antibiotic prophylaxis, post-operative infections

## Abstract

The impact of third-generation cephalosporin-resistant Enterobacterales (3GCR-E) rectal colonization in the development of subsequent infection after surgery is controversial. In particular, there is a lack of data in the context of hepato-pancreato-biliary (HPB) surgery. The objective of this study was to assess the prevalence of 3GCR-E intestinal carriage among patients undergoing elective HPB resection surgery and its impact on the incidence and etiology of surgical site infections (SSIs). This retrospective cohort study (January 2016–December 2022) was performed at Valme University Hospital (Seville, Spain). The inclusion criteria included (i) 18 years of age or older, (ii) undergoing elective HPB resection surgery, and (iii) availability of a periprocedural surveillance rectal swab culture to detect 3GCR-E. The prevalence of 3GCR-E intestinal carriage at elective HPB resection surgery was assessed, as well as SSI incidence at 30 days and possible associated factors. Two hundred nine patients were included. Eleven (5.3%) patients were colonized by 3GCR-E at baseline. According to 3GCR-E carriage status, 6 (55%) of the carriers developed SSI, whereas this occurred in 50 (25%) of non-carriers (*P* = 0.033). Likewise, the rates of SSI caused specifically by 3GCR-E were 83% (5 of 6) in 3GCR-E carriers and 6% (3 of 50) in non-carriers (*P* < 0.001). After multivariate analyses, 3GCR-E colonization at the time of surgery was identified as an independent predictor for developing SSI (adjusted odds ratio 4.63, 95% confidence interval: 1.177–18.232, *P* = 0.028). Despite a low prevalence of 3GCR-E intestinal carriage at surgery, 3GCR-E rectal colonization is associated with a higher risk of SSI among patients undergoing elective HPB resection surgery, with most SSIs being caused by the colonizing bacteria.

**IMPORTANCE:**

In this Spanish retrospective cohort study, previous 3GCR-E rectal colonization was associated with a higher risk of SSI after hepato-pancreato-biliary resection surgeries. Most of SSIs were caused by the colonizing bacteria, suggesting a rationale for adapted perioperative antibiotic prophylaxis in known 3GCR-E colonized patients.

## INTRODUCTION

Antimicrobial resistance (AMR) is currently considered one of the top 10 global public health threats worldwide, as it has been associated with increased morbidity, mortality (1), and excess medical costs (2). In 2017, the World Health Organization (WHO) published a list of antibiotic-resistant “priority pathogens” in a bid to promote research and development of new antibiotics. The most critical group included various *Enterobacteriaceae* resistant to third-generation cephalosporins. In fact, according to data published in 2023 by the European Antimicrobial Resistance Surveillance Network, the proportions of *Escherichia coli* and *Klebsiella pneumoniae* resistant to third-generation cephalosporins were 13.8% and 34.3%, respectively (3).

Rectal colonization by third-generation cephalosporin-resistant Enterobacterales (3GCR-E) has been suggested as a potential risk factor of subsequent infection from the colonizing strain, particularly in high-risk environments as intensive care (4) or hematology units (5). However, the precise magnitude of this risk remains unclear.

In the surgical setting, there is a lack of robust data regarding the role of previous multidrug-resistant Gram-negative bacteria (MDR-GNB) rectal colonization on post-surgical outcomes. The primary evidence comes from urologic surgery, in which a relationship between baseline carriage of fluoroquinolone-resistant pathogens and an increased risk of post-transrectal prostate biopsy infections has been shown (6, 7). Data on abdominal surgery are mainly limited to liver transplantation (LT) and colorectal surgery (8–11). It involves few observational studies along with a systematic review and meta-analysis of these studies carried out by Righi et al. (12), showing an increased risk of post-operative 3GCR-E infections, including surgical site infections (SSIs), in 3GCR-E colonized patients compared with non-colonized ones. If an increased risk in colonized patients is confirmed, an adapted perioperative antibiotic prophylaxis (PAP) based on previous rectal culture results might be considered. In this regard, the European Society of Clinical Microbiology and Infectious Diseases/European Committee on Infection Control guidelines recommend 3GCR-E rectal screening and subsequent targeted PAP only before colorectal and LT surgery, with low certainty of evidence (13), while the last WHO guidelines did not formulate a recommendation due to the lack of evidence (14).

On the other hand, there are high-risk surgical procedures other than LT or colorectal surgery, such as hepato-pancreato-biliary (HPB) resection surgery, in which the potential impact of prior 3GCR-E rectal carriage may vary. It is not known if 3GCR-E presence in colorectal flora correlates with that found at the biliary tract and its role in post-procedural infectious complications. For the above-mentioned reasons, the aim of this study was to assess the prevalence of 3GCR-E intestinal carriage among patients undergoing elective HPB resection surgery and its impact on the incidence and etiology of SSI.

## MATERIALS AND METHODS

### Design and patients

This was a retrospective cohort study including all patients attended at the Valme University Hospital (Seville, Spain) from January 2016 to December 2022 who fulfilled the following criteria: (i) 18 years of age or older; (ii) undergoing elective HPB resection surgery, including hepatobiliary resection, pancreatoduodenectomy–Whipple’s resection, and distal pancreatectomies; and (3) availability of a periprocedural surveillance rectal swab culture to detect MDR-GNB, carried out 24 h before or after surgery as part of intensive care unit (ICU) surveillance protocol at admission.

### Patient management

At our institution, all patients who undergo one of the HPB procedures described above are admitted to the ICU during the first 24–48 h after surgery. Since 2016, all patients admitted to the ICU are screened for MDR-GNB colonization at admission, as part of institutional surveillance protocol and following the national recommendations from the “Zero Resistance” project of the Spanish Society of Intensive Care Medicine and Coronary Care Units (15).

The two primary surgical procedures were hepatectomy and pancreatectomy. Regarding hepatectomy, two main types of surgeries were performed: anatomic hepatectomy, which involved the resection of one or several anatomic segments (segmentectomy, bisegmentectomy, or right or left hepatectomy), and a non-anatomic one, usually an atypical resection with a narrower focus on tumor removal. In these hepatic surgeries, there is no manipulation of the bile duct, except those carried out within the context of tumors at the biliary confluence (Klatskin tumors), in which a biliary–enteric anastomosis is performed concomitantly.

On the other hand, two major types of pancreatic surgeries were carried out: those that involved resection of the head of the pancreas, whose paradigm was pancreatoduodenectomy or Whipple’s resection; and those that focused on resections of the pancreatic tail–body, mainly distal pancreatectomies with or without splenic preservation. Since 2018, cultures of bile samples obtained during surgery were conducted in most of Whipple’s resections at the discretion of the surgical team.

PAP regimens were administered following local antibiotic policy recommendations for HPB surgeries and in accordance with national guidelines in force during the study period (16); namely, the recommended PAP regimen consisted of a single dose of 2-g cefazolin or 2-g amoxicillin–clavulanic acid, administered intravenously between 120 and 30 min before the incision. Piperacillin–tazobactam (4.5 g) was given in the case that a preoperative biliary drainage had been done, according to surgical team criteria, in line with the recommendations of other surgical groups (17). An additional intraoperative dose was given after 3 h of surgery or when there was significant bleeding (>1500 mL) (16).

Patients were managed according to the criteria of the ICU and/or general surgery ward’s caring physician, including the indication and selection of antibiotic therapy in case of post-operative infectious complications, which followed the local antibiotic policy recommendations at our center.

### Data collection

Patients undergoing elective HPB resection surgery during the study period were identified from a specific database of the general surgery unit, in which all patients undergoing elective HPB resection surgery were prospectively included since 2016. All electronic clinical records were retrospectively reviewed for this study to ascertain that rectal swab cultures to detect MDR-GNB were done 24 h before surgery or up to 24 h after. Patients were followed up to 30 days after the index surgery.

Demographic variables, comorbidities including Charlson Age-Adjusted Comorbidity Index, history of hospitalization in the previous year, antibiotics exposures within the previous 3 months, as well as the need for and method of preoperative biliary drainage were collected. Regarding surgery, surgical procedure (hepatobiliary resection, pancreatoduodenectomy, or distal pancreatectomy), operative approach (laparoscopic, primary open, or converted procedure), operating room time, and PAP regimen were assessed.

Post-operative complications were registered and classified according to the Clavien–Dindo system (18), focusing mainly on the assessment of hospital-acquired infections (HAIs), including SSI, pneumonia, urinary tract infection, primary bloodstream infection, catheter-related bloodstream infection, and fever without source, and its management. According to the Centers for Disease Control and Prevention definition, SSIs were defined as a superficial or deep/organ space infection (19). SSI microbiological etiology was described based on organisms identified from an aseptically obtained specimen by a culture-based microbiological testing method.

### Diagnostic procedures

Surveillance rectal swab samples were cultured in chromogenic culture media for the detection of 3GCR-E (chromID ESBL Agar, bioMérieux) and carbapenemases (chromID TMCARBA, Becton Dickinson). MacConkey plates seeding (Becton Dickinson) was used as a growth control. Using this approach extended spectrum β-lactamase (ESBL)- or AmpC-producing *Enterobacteriaceae*, we found that carbapenem-resistant Enterobacterales, *Stenotrophomonas maltophilia*, MDR *Acinetobacter baumannii*, and MDR *Pseudomonas aeruginosa* can be identified. In case of growth on the carbapenemases detection plate, a colorimetric test (β-CARBA test, Bio-Rad) was also carried out with further identification of bacterial isolates being performed by Matrix-Assisted Laser Desorption/Ionization–Time-Of-Flight mass spectrometry (Bruker) and carbapenemase characterization through inhibitor disks or immunochromatography, such as NG-Test CARBA 5 (Labymed S.A.). In surveillance cultures, antibiotic susceptibility testing was not routinely performed, following the Spanish Society of Infectious Diseases and Clinical Microbiology recommendations (20).

Upon any clinical suspicion of SSI, samples were aseptically collected whether from deep soft tissue incision or from fluid or tissue in the organ/space, depending on whether it was superficial or deep SSI, respectively. The quality of these specimens was assessed by Gram stain, and no epithelial cells were observed in any of them. Therefore, all surgical wound specimens were cultured. These samples were inoculated on different agar plates for aerobic (blood agar, chocolate agar, and MacConkey agar) and anaerobic culture (Schaedler agar and thioglycolate broth).

All isolates were screened for ESBL, AmpC, and/or carbapenemase production by double-disk synergy test, cloxacillin synergy test, and combination disk testing, respectively, according to the European Committee on Antimicrobial Susceptibility Testing guidelines. For the genotypic confirmation of the presence of ESBL and carbapenemase, genes were characterized by polymerase chain reaction and further sequencing in a reference center.

### Statistical analysis

The primary outcome variable of the study was the prevalence of 3GCR-E intestinal carriage at elective HPB resection surgery. Secondary outcome variables were the incidence of post-operative HAI at 30 days and the incidence of SSI at 30 days.

Descriptive analyses included the description of the microbiological etiology of 3GCR-E colonization and post-operative infectious episodes. Continuous variables were expressed as medians (quartile 1–quartile 3 and categorical variables as frequencies (percentages).

The factors associated with the primary and secondary outcome variables were identified by univariate and multivariate analyses. For SSI, 3GCR-E colonization at baseline was included in the analyses of potential post-operative infection risk factors. Continuous variables were compared by means of the Student *t*-test or the Mann–Whitney *U* test, depending on the normality tests. The χ^2^ and Fisher tests were used for comparisons between categorical variables.

Variables with a *P* value less than 0.1 on univariate analyses were entered in logistic regression multivariate analyses. Differences were considered statistically significant for *P* values of 0.05 or less. Adjusted odds ratios (AORs) and the respective 95% confidence intervals (CIs) were calculated. Data were analyzed using IBM SPSS version 24.0 (IBM Corporation, Somers, NY, USA).

## RESULTS

### Features of the study population

**Fig 1:**
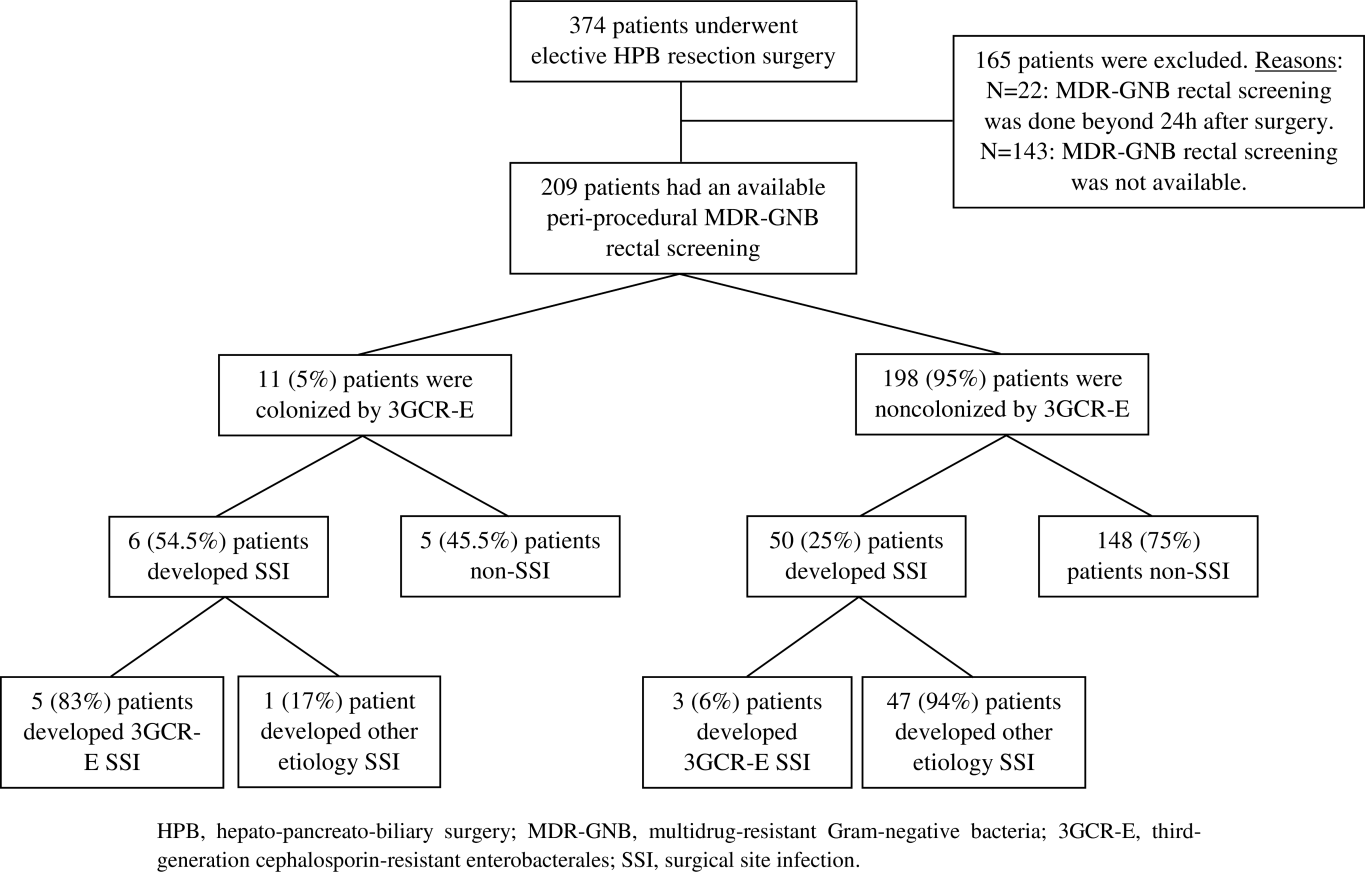
Study flow diagram.

During the study period, 374 adult patients underwent elective HPB resection surgery at our center. Of these patients, 209 who had an available periprocedural MDR-GNB rectal surveillance culture comprised the study population (Fig. 1). The main baseline characteristics of these patients are summarized in Table 1. Most of surgeries (89%) were oncological surgeries. Seventy-seven (37%) patients had antibiotic exposure within the previous 3 months, with up to 34 patients receiving two or more antibiotic courses.

**TABLE 1:** Main baseline characteristics of the study population (*N* = 209)^*i*^

CharacteristicS, *n* (%)	Value
Age (years)^*a*^	66 (56–74)
Male sex	135 (65)
Diabetes mellitus	48 (23)
Immunosuppression^*b*^	60 (29)
Active cancer	186 (89)
Pancreatic cancer	56 (27)
Cholangiocarcinoma	17 (8)
Carcinoma of the ampulla of Vater	8 (4)
Colorectal cancer liver metastases	75 (36)
Others^*c*^	30 (14)
Age-adjusted Charlson Comorbidity Index^*a*^	6 (4–10)
Hospitalization in the previous year	137 (66)
Antibiotics within the previous 3 months	77 (37)
Penicillins and/or amoxicillin–clavulanic acid^*d*^	37/134 (28)
Cephalosporins^*d*^	22/134 (16)
Piperacillin–tazobactam^*d*^	22/134 (16)
Quinolones^*d*^	16/134 (12)
Preoperative biliary drainage within the previous 3 months	55 (26)
Percutaneous transhepatic biliary drainage	14 (7)
Endoscopic biliary drainage	37 (18)
Surgical drainage	4 (2)
Type of surgery	
Anatomic hepatectomies^*e*^	74 (35)
Non-anatomic hepatectomies	47 (23)
Whipple’s resection	75 (36)
Distal pancreatectomy	13 (6)
Operative approach	
Laparoscopic surgery	35 (17)
Primary open surgery	159 (76)
Converted surgery	15 (7)
Operating room time (min)^*a*^^,*f*^	300 (250–360)
PAP regimen^*g*^	
Cefazolin	148 (73)
Amoxicillin–clavulanic acid	10 (5)
Piperacillin–tazobactam	35 (17)
Others^*h*^	11 (5)

^*a*^ Median (Q1–Q3).

^*b*^ Immunosuppression: HIV infection with CD4 <200 cells/microL, active chemotherapy, and immunosuppressive treatment.

^*c*^ Gallbladder cancer, hepatocellular carcinoma, duodenal cancer, and liver metastases from cancers other than colorectal cancer.

^*d*^ Referred to overall courses of antibiotics received, considering there were patients who took up to five different antibiotic courses.

^*e*^ Concomitant biliary–enteric anastomosis was carried out in 3 out of 74 anatomic hepatectomies performed in the context of tumors at the biliary confluence (Klatskin tumors).

^*f*^ Operating room time available in 206 patients.

^*g*^ PAP regimen available in 204 patients.

^*h*^ Regimens with aminoglycosides and antianaerobic antibiotics due to history of penicillin allergy.

^*i*^ PAP, perioperative antibiotic prophylaxis.

Fifty-five (26%) patients needed a biliary drainage within the 3 months before HPB surgery, mainly due to malignant biliary obstruction. Hepatobiliary resection was the most common surgical procedure (58%), while the primary open surgical approach was the most frequently used approach (76%) (Table 1).

### Prevalence of 3GCR-E colonization and its correlation with bile cultures at the index surgery

3GCR-E colonization was found in 11 (5.3%) patients (*n* = 8 ESBL-producing *E. coli*, *n* = 1 ESBL-producing *K. pneumoniae*, *n* = 2 AmpC-producing *Enterobacter cloacae*). Receiving antibiotics within the previous 3 months was more frequent in 3GCR-E colonized patients [8 (77%) out of 11 vs 69 (35%) out of 198 non-3GCR-E colonized patients, *P* = 0.011]. Table S1 summarizes the univariate analysis performed to identify the risk factors of 3GCR-E colonization.

Colonizing 3GCR-E antibiotic susceptibility was available in 4 out of 11 3GCR-E carriers. The PAP regimen was effective according to the antibiogram in only one case, in which piperacillin–tazobactam was used. Overall, 7 out of 11 colonized patients received a cefazolin-based PAP that had no activity against 3GCR-E.

Cultures of bile specimens at the index surgery were collected in 56 (75%) out of 75 elective pancreatoduodenectomies. Bile cultures were positive in 45 (80%) of them, from which 3GCR-E was detected in five (9%) patients (one 3GCR-E rectal carrier and four non-carriers) (Table S2). Among the four patients who were 3GCR-E bile duct carriers but did not have 3GCR-E rectal colonization, only one developed SSI, which was polymicrobial, involving wild-type *K. pneumoniae*, wild-type *Citrobacter freundii*, and *Enterococcus faecalis*. The fifth patient, who had 3GCR-E colonizing both the biliary tree and the rectum, developed 3GCR-E SSI.

On the other hand, four 3GCR-E colonized patients underwent an elective pancreatoduodenectomy. Bile cultures were available in three of them. In one patient, the same colonizing strain was isolated in the bile culture, whereas in the other two patients, different microorganisms were identified (Table S2).

### Incidence and microbiological etiology of post-operative infections

Among the 209 patients comprising the study population, 73 (35%) developed a total of 88 HAI episodes within 30 days after HPB resection surgery. Figure S1 summarizes the specific sources of these infections.

Fifty-six (27%) out of the 209 patients included in the study developed SSI, of which 15 (27%) were superficial and 41 (73%) were deep SSIs. Among the 56 SSI episodes, 28 (50%) were polymicrobial infections. In 5 (9%) cases of the 56 SSIs, cultures from clinical samples were negative, all of which were collected under empirical antibiotic therapy. Microorganisms isolated from representative samples of these SSI episodes are described in Table 2.

**TABLE 2:** Description of the 93 microorganisms isolated from representative samples of 56 SSI episodes^*a*^*^,^*^*b*^

Microorganism, *n* (%)	Value
Gram-positive bacteria	
*Staphylococcus aureus*	2 (2)
*Streptococcus* spp.	3 (3)
*Enterococcus faecalis*	7 (8)
*Enterococcus faecium*	9 (10)
*Enterococcus gallinarum*	1 (1)
Gram-negative bacteria	
*Escherichia coli*	13 (14)
ESBL-producing *E. coli*	3 (3)
*Klebsiella pneumoniae*	11 (12)
ESBL-producing *K. pneumoniae*	1 (1)
*Klebsiella oxytoca*	2 (2)
ESBL-producing *K. oxytoca*	1 (1)
*Enterobacter cloacae*	4 (4)
AmpC-β-lactamase-producing *E. cloacae*	3 (3)
*Enterobacter aerogenes*	3 (3)
*Citrobacter freundii*	3 (3)
*Serratia marcescens*	3 (3)
*Proteus mirabilis*	2 (2)
*Morganella morganii*	4 (4)
*Pseudomonas aeruginosa*	8 (9)
Anaerobic microorganisms	7 (8)
*Candida* sp.	3 (3)

^*a*^ There were 93 microorganisms recovered from 56 patients with SSI.

^*b*^ ESBL, extended spectrum β-lactamase; SSI, surgical site infection.

### 3GCR-E colonization and risk of SSI

**Fig 2:**
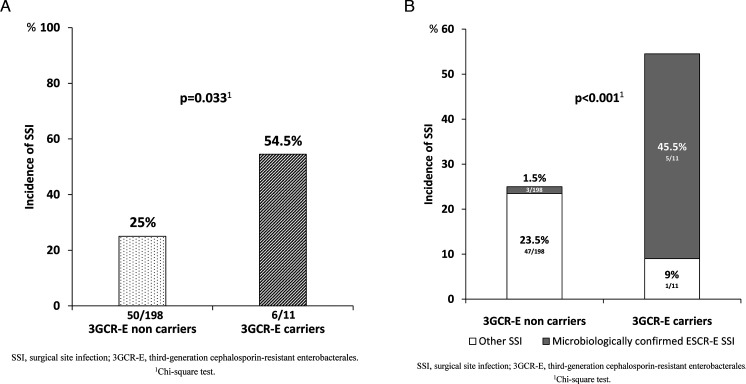
(A) Incidence of SSI according to 3GCR-E carriage status. (B) Incidence of SSI and microbiological etiology according to 3GCR-E carriage status.

According to 3GCR-E carriage status, 6 (54.5%) of the carriers showed SSI, whereas this occurred in 50 (25%) of the non-carriers (*P* = 0.033) (Fig. 2A). Likewise, rate of SSI caused specifically by 3GCR-E was 45.5% (5 of 11) in 3GCR-E carriers and 1.5% (3 of 198) in non-carriers (*P* < 0.001) (Fig. 2B). Indeed, when assessing the microbiological etiologies of SSI, 5 (83%) out of 6 colonized patients who developed SSI were due to 3GCR-E compared to 3 (6%) out of 50 non-colonized ones with SSI.

### Independent risk factors of SSI

Table 3 summarizes univariate and multivariate predictors of SSI development. Twenty-two (40%) out of 55 patients who underwent biliary drainage within the previous 3 months of HPB resection surgery developed SSI, whereas this occurred in 34 (22%) out of 154 patients who did not require preoperative biliary drainage (*P* = 0.01). Regarding the type of surgery, SSI was more frequent after pancreatic surgeries than after hepatectomies [30 of 88 (34%) vs 26 of 121 (22%), *P* = 0.042].

**TABLE 3:** Univariate and multivariate analyses of the predictors for developing SSI^*g*^

Factor	*n*/*N* (%)	*P* bivariate	Adjusted odds ratio (95% CI)	*P* multivariate
Age (years)				
<65	19 (21)			
≥65	37 (32)	0.075	1.030 (0.997–1.063)^*a*^	0.074
Sex				
Women	19 (26)			
Men	37 (27)	0.787	–	**–**
Diabetes mellitus				
No	44 (27)			
Yes	12 (25)	0.749	–	–
Immunosuppression^*b*^				
No	44 (30)			
Yes	12 (20)	0.159	–	–
Age-adjusted Charlson Comorbidity Index				
<10	45 (31)			
≥10	11 (17)	0.03	0.949 (0.805–1.120)^*a*^	0.537
Hospitalization in the previous year				
No	19 (26)			
Yes	37 (27)	0.924	–	–
Preoperative biliary drainage within the previous 3 months				
No	34 (22)			
Yes	22 (40)	0.010^*c*^	1.632 (0.656–4.064)	0.293
Antibiotics within the previous 3 months				
No	26 (20)			
Yes	30 (39)	0.002^*c*^		
3GCR-E rectal screening				
Negative	50 (25)			
Positive	6 (55)	0.033	4.632 (1.177–18.232)	0.028
Active cancer				
No	10 (43)			
Yes	46 (25)	0.055	0.381 (0.110–1.315)	0.127
Type of surgery				
Hepatectomy	26 (22)			
Pancreatectomy	30 (34)	0.042	1.060 (0.422–2.659)	0.902
Operative approach				
Laparoscopic surgery	5 (14)			
Open surgery	51 (29)	0.067	1.980 (0.681–5.755)	0.209
Operating room time ≥5 h^*d*^				
No	18 (20)			
Yes	36 (31)	0.074	2.511 (1.148–5.493)	0.021
Appropriate PAP^*e*^^,*f*^				
No	16 (30)			
Yes	39 (26)	0.505	0.469 (0.211–1.044)	0.064

^*a*^ Considered as a continuous variable in the multivariate analysis.

^*b*^ Immunosuppression: HIV infection with CD4 <200 cells/microL, active chemotherapy, immunosuppressive treatment.

^*c*^ Collinearity between preoperative biliary drainage within the previous 3 months and previous antibiotic therapy was detected, which precluded the inclusion of both parameters in multivariate models. This led to the exclusion of antibiotics within the previous 3 months in the final model.

^*d*^ Operating room time available in 54 of 56 patients who developed SSI.

^*e*^ PAP regimen available in 55 of 56 who developed SSI.

^*f*^ Appropriate PAP: if it was administered according to local antibiotic policy recommendations for hepato-pancreato-biliary surgeries, including an additional intraoperative antibiotic dose when indicated.

^*g*^ 3GCR-E, third-generation cephalosporin-resistant Enterobacterales; PAP, perioperative antibiotic prophylaxis.

After multivariate analyses, the independent predictors for developing SSI were 3GCR-E colonization at the time of surgery (AOR 4.632, 95% CI: 1.177–18.232, *P* = 0.028) and operating room time of ≥5 h (AOR 2.511, 95% CI: 1.148–5.493, *P* = 0.021) (Table 3).

## DISCUSSION

Our study shows that previous 3GCR-E rectal colonization is associated with a higher risk of SSI among patients undergoing elective HPB resection surgery. According to our data, in 3GCR-E colonized patients, SSIs after these surgeries are predominantly caused by the colonizing bacteria. Ineffective coverage by empiric PAP regimens in 3GCR-E colonized patients could explain these findings.

There are very few observational studies and no randomized controlled trials assessing the role of previous colonization by rectal 3GCR-E in the risk of SSI after abdominal surgery, most of them conducted in patients undergoing LT or colorectal surgery (13). Among studies on LT patients, there are only two relevant studies in which the 3GCR-E colonization rates were 16% (9) and 13% (10). In both studies, the proportion of 3GCR-E infections was higher among pre-LT colonized patients compared to non-carriers, although only one of them provided data on the specific type of infection, which included 11 SSI cases (10). Regarding colorectal surgery, the most robust study, conducted by Dubinsky-Pertzov et al. (11), included 3,600 patients screened for 3GCR-E colonization prior to surgery, of which 14% were carriers. In spite of higher rates of SSI in 3GCR-E colonized patients, SSI caused specifically by 3GCR-E were scarce, although somewhat more common in colonized patients [8% (17 of 222) in carriers vs 2% (7 of 440) among non-carriers]. To date, published data in surgical settings other than LT or colorectal surgery are scarce. There are two prospective cohort studies encompassing different types of gastrointestinal and gynecological surgeries, with 3GCR-E colonization rates ranging from 17.5% (21) to 36% (22). Once again, there was a higher proportion of SSI among colonized patients, although drawing definitive conclusions is challenging due to their small sample size, high heterogeneity in terms of type of surgery and prophylaxis, as well as limited SSI cases due to 3GCR-E.

Regarding HPB surgery, De Pastena et al. performed a prospective, non-randomized, non-blinded, interventional study to evaluate the effectiveness of piperacillin–tazobactam (vs ampicillin–sulbactam) as PAP in patients undergoing elective pancreatic surgery for a periampullary tumor (23). Forty-seven (14%) 3GCR-E carriers in the baseline phase and 29 (10%) in the intervention phase were identified. In the baseline period, colonized patients had a higher rate of HAI and superficial SSI, but no differences were found when deep or organ/space SSIs were compared among periods. Besides, post-surgical infection etiology was not detailed in 3GCR-E carriers. Consequently, it is not known whether 3GCR-E colonized patients were also infected by 3GCR-E strains nor whether the lower post-surgical infection rate observed during the interventional phase was due to the use of piperacillin–tazobactam as PAP.

To our knowledge, this is the first study that has assessed the impact of 3GCR-E intestinal carriage on the risk of SSI in patients undergoing elective HPB resection surgery. According to our data, the incidence of SSI after these types of surgeries is higher in 3GCR-E colonized patients, with SSI episodes mainly caused by the colonizing strain. Although there is a risk of bias inherent to the retrospective design of our study, the association between 3GCR-E colonization and a higher risk of SSI was independent of other factors, including many potential confounders. This suggests that rectal 3GCR-E colonization could truly contribute to a higher risk of SSI and is not merely a confounding factor. The reason why 3GCR-E intestinal colonization might increase the risk of SSI after surgery involving the biliary tract is not known. Our hypothesis is that the presence of 3GCR-E in colorectal flora could partially correlate with that found at the biliary tract, which is ultimately the responsible flora of SSI in HPB surgeries. In this sense, one out of three 3GCR-E carriers with an available surgical bile sample showed a correlation between bile culture and rectal swab culture. Unfortunately, routine bile culture at the index surgery has not been uniformly performed in our cohort, as such a procedure has not been endorsed by previous international guidelines (24). Assuming that some grade of concordance of the rectal flora with that colonizing the biliary tract exists, the higher risk of SSI among 3GCR-E carriers would probably not be due to the fact of being 3GCR bacteria *per se* but due to a higher probability of receiving a non-active PAP regimen. Thus, a recent multicenter, randomized clinical trial showed that the use of broad-spectrum antibiotic such as piperacillin–tazobactam as PAP in patients undergoing open pancreatoduodenectomy reduced post-operative SSI compared with standard of care (25). Indeed, in a post hoc analysis, it was suggested that this reduction in post-operative SSI in patients receiving piperacillin–tazobactam as PAP compared with cefoxitin may be mediated by the presence of certain biliary bacteria that are resistant to cefoxitin, notably *Enterobacter* spp. and *Enterococcus* spp (26). However, extrapolating the results of this trial in a one-size-fits-all approach is also problematic and could led to unnecessary antibiotic coverage and a further increase in AMR burden. Keeping in mind that PAP effectiveness presupposes that the agent used needs to be active against the pathogens likely to cause SSI, we found that a more targeted approach would be more desirable than a broader indication of broad-spectrum PAP. Our results suggest that when it is known that the patient is an MDR-GNB carrier (whether from a preoperative bile culture or a previous 3GCR-E rectal surveillance culture), it seems prudent to modify PAP to include an active agent, either alone or added to the standard regimen (27–29). Although in some studies adopting a rectal culture-based PAP strategy led to better outcomes (30, 31), universal 3GCR-E intestinal carriage screening prior to surgery to perform targeted PAP is still not formally recommended by most guidelines (13, 14). Alternatively, future research should evaluate if surveillance of high-risk patients could be cost effective.

This study has some limitations that must be acknowledged. First, the single-center retrospective design of the study, in which only patients screened at admission to ICU for MDR-GNB colonization were enrolled, limits the generalizability of the findings. Besides, a proportion of patients who underwent elective HPB resection surgery during the study period were excluded as 3GCR-E colonization surveillance upon admission to ICU had not been performed, which was likely due to the impact of the coronavirus disease 2019 pandemic on standard practices in recent years. Second, the lack of genotypic characterization of 3GCR-E prevents us from conclusively assuming that the colonizing strain and the infection-causing strain were the same, although this is a common limitation in real-world studies as molecular typing is not typically performed routinely. Third, the 3GCR-E intestinal carriage rate in our sample, which resembled resistance rates in our region, was relatively low when compared to other settings. Thus, the results of our work, which would apply for regions with similarly low rates, should be confirmed in areas with a higher burden of 3GCR-E colonization. Fourth, our study is limited to HPB resection surgeries, and similar results might not be assumed in other hepatobiliary and pancreatic surgeries that do not involve manipulation of the bile duct. A final limitation is the lack of surgical bile samples in a proportion of cases, hindering the ability to establish a definite correlation between rectal and biliary tract flora. In spite of this, there is a growing body of evidence supporting that the increase in antibiotic resistance, particularly the spread of ESBL-producing *Enterobacteriaceae*, impacts on gram-negative microorganisms colonizing the biliary tract and could potentially reduce the effectiveness of antibiotic agents traditionally recommended as PAP (32–34). On the other hand, several strengths of our study should also be noted. This is the first study with the largest sample size of individuals undergoing HPB resection surgery, including data about 3GCR-E intestinal carriage status prior to surgery and assessing SSI specifically caused by 3GCR-E as an outcome. Besides, as previously discussed, the multivariate analyses included potential confounding factors that have not been taken into account in other studies. Thus, another independent predictor for developing SSI in our study was operating room time of ≥5 h, in line with what is reported in the literature (35).

In summary, 3GCR-E colonization at the time of surgery was an independent risk factor for developing SSI after an elective HPB resection surgery in a low-prevalence setting. As non-active PAP could explain these findings, further research should evaluate the effectiveness of a strategy of 3GCR-E carriage screening prior to these surgeries with subsequent targeted PAP. According to our results, it seems reasonable to modify PAP to include an active agent in patients already known to be colonized with 3GCR-E undergoing HPB resection surgery.

## SUPPLEMENTAL MATERIAL

Supplemental material spectrum.00878-24-s0001.pdf Tables S1 and S2; Fig. S1.

ASM does not own the copyrights to Supplemental Material that may be linked to, or accessed through, an article. The authors have granted ASM a non-exclusive, world-wide license to publish the Supplemental Material files. Please contact the corresponding author directly for reuse.
