# Supplementary material for: Role of rectal colonization by third-generation cephalosporin-resistant Enterobacterales on the risk of surgical site infection after hepato-pancreato-biliary surgery
Source: Microbiol Spectr. 2024 Sep 24;12(11):e00878-24. doi: 10.1128/spectrum.00878-24 (PMC11537004; doi:10.1128/spectrum.00878-24)
Supplement: Supplemental material — Tables S1 and S2; Fig. S1. [file spectrum.00878-24-s0001.pdf]

Supplementary Table 1. Univariate analysis of the predictors of 3GCR-E colonization

Factor	3GCR-E colonized patients (n=11), n (%)	Non 3GCR-E colonized patients (n=198), n (%)	p value
Age ≥65 years	6 (54.5)	111 (56)	0.922
Male sex	5 (45.5)	130 (66)	0.173
Diabetes mellitus	1 (9)	47 (24)	0.261
Immunosuppression[a]	3 (27)	57 (29)	0.914
Age-adjusted Charlson Comorbidity Index ≥10	2 (18)	63 (32)	0.342
Hospitalization in the previous year	6 (54.5)	131 (66)	0.430
Antibiotics within the previous 3 months	8 (73)	69 (35)	**0.011**
Active cancer	10 (91)	176 (89)	0.835
Type of cancer			0.684[b]
Pancreatic cancer	4 (40)	52 (29.5)	
Colorectal cancer liver metastases	4 (40)	71 (40)	
Biliary tract cancer[c]	0	31 (18)	
Others[d]	2 (20)	22 (12.5)	
Preoperative biliary drainage within the previous 3 months	5 (45.5)	50 (25)	0.139

3GCR-E, third-generation cephalosporin-resistant enterobacterales.

[a]Immunosuppression: HIV infection with CD4 < 200 cells/microL, active chemotherapy, immunosuppressive treatment; [b]Linear-by-linear association test; [c]Biliary tract cancer: gallbladder cancer, cholangiocarcinoma and carcinoma of the ampulla of Vater; [d]Others: hepatocellular carcinoma, duodenal cancer and liver metastases from other than colorectal cancer.

Supplementary Table 2. Description of the 95 microorganisms isolated from bile samples obtained during Whipple's resections in 56 patients according to rectal colonization status

Microorganism, no. (%)	Value
**3GCR-E rectal colonized patients (N=3; 8 microorganisms)**	
Negative cultures	0
Gram-positive bacteria	
Enterococcus faecium	1 (13)
Gram-negative bacteria	
**ESBL-producing *Escherichia coli***	1 (13)
Klebsiella pneumoniae	1 (13)
Enterobacter cloacae	2 (25)
Morganella morganii	1 (13)
Pseudomonas aeruginosa	1 (13)
Candida albicans	1 (13)
**3GCR-E rectal noncolonized patients (N=53; 87 microorganisms)**	
Negative cultures	11
Gram-positive bacteria	
Streptococcus spp.	9 (10)
Enterococcus faecalis	6 (7)
Enterococcus faecium	7 (8)
Enterococcus avium	1 (1)
Clostridium perfringens	2 (2)
Gram-negative bacteria	
Escherichia coli	7 (8)
Klebsiella pneumoniae	7 (8)
Klebsiella oxytoca	7 (8)
Enterobacter cloacae	13 (15)
**AmpC-producing *E. cloacae***	1 (1)
Enterobacter aerogenes	1 (1)
**AmpC-producing *E. aerogenes***	2 (2)
Citrobacter freundii	3 (3)
**AmpC-producing *C. freundii***	1 (1)
Other enterobacterales[1]	9 (10)
Pseudomonas aeruginosa	3 (3)
Anaerobic microorganisms	7 (8)
Candida tropicalis	1 (1)

3GCR-E, third-generation cephalosporin-resistant enterobacterales; ESBL, extended spectrum β-lactamase.

A total of 95 isolates from bile samples collected in 56 patients who underwent elective Whipple's resection, distributed as follows: 8 microorganisms isolated in the three 3GCR-E rectal colonized patients and 87 microorganisms isolated in the fifty-three 3GCR-E noncolonized patients. [1]Other enterobacterales: *Serratia marcescens* (2), *Proteus mirabilis* (1), *Morganella morganii* (2), *Citrobacter amalonaticus* (1), *Citrobacter braakii* (1), *Citrobacter koseri* (1), *Hafnia alvei* (1).

Supplementary Figure 1. Forms of hospital-acquired infection

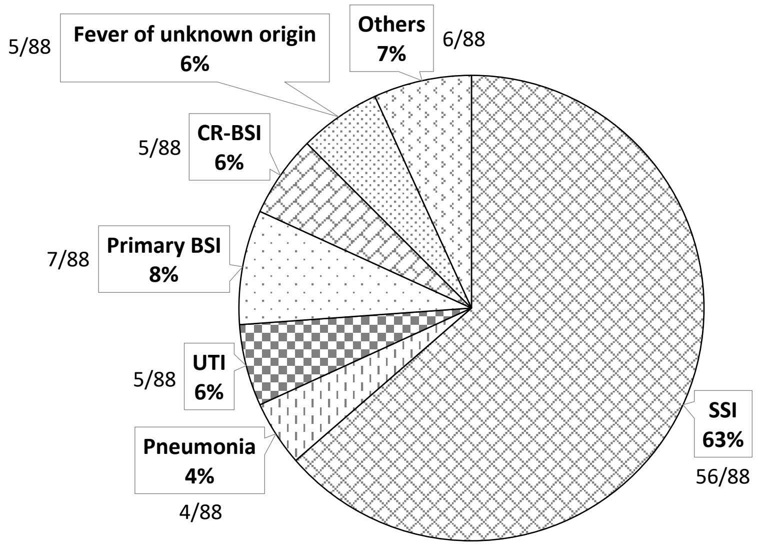

SSI, surgical site infection; UTI, urinary tract infection; BSI, bloodstream infection; CR-BSI, catheter-related bloodstream infection.

Referred to 88 infectious events in 73 patients. Among 56 patients who developed SSI, 11 had a second infectious episode and 4 had three different hospital-acquired infections episodes within 30 days after surgery.
